# Efficacy and Safety of the L-Scar Technique in Breast Surgery: A Systematic Review and Meta-Analysis

**DOI:** 10.1007/s00266-026-05869-1

**Published:** 2026-05-11

**Authors:** Pedro Lucas Machado Magalhães, Bernardo Gabriele Collaco, Milena Gonçalves Guerreiro, Anderson Matheus Pereira da Silva, Luciano Falcão, Dillan Cunha Amaral, Rafael Andrade Sampaio Silva, Mariana Letícia de Bastos Maximiano, Isabela Azevedo de Almeida, Yasmin Picanço Silva, Bernardo Gonçalves Bastian Pinto, Adekunle I. Elegbede

**Affiliations:** 1 Faculty of Medicine, Institute of Medical Education, Angra dos Reis, RJ Brazil; 2 Division of Plastic Surgery, Mayo Clinic, 4500 San Pablo Rd S, Jacksonville, FL 32224 USA; 3 Faculty of Medicine, University of Araraquara, Araraquara, SP Brazil; 4 Division of Biostatistics and Clinical Epidemiology, MemoryHub, Cognitive and Neurodegenerative Disorders Research Group, São Paulo, SP Brazil; 5 Faculty of Medicine, Bahiana School of Medicine and Public Health, Salvador, BA Brazil; 6 Faculty of Medicine, Federal University of Rio de Janeiro, Rio de Janeiro, RJ Brazil; 7 Faculty of Medicine, Federal Fluminense University, Niterói, RJ Brazil; 8 Faculty of Medicine, Federal University of Juiz de Fora, Juiz de Fora, MG Brazil; 9 Healthcare Institution of South Iceland, Selfoss, Iceland; 10 Department of Plastic Surgery, Barata Ribeiro Hospital, Rio de Janeiro, RJ Brazil

**Keywords:** Mammaplasty, Breast, Meta-analysis, Systematic review, Cicatrix, L-scar technique

## Abstract

**Background:**

The L-scar technique has emerged as a promising alternative in breast surgery, aiming to reduce visible scarring while preserving aesthetics and minimizing complications. Despite growing clinical interest, comprehensive evaluations of its outcomes remain scarce.

**Methods:**

We conducted a systematic review and meta-analysis following PRISMA and Cochrane guidelines. Studies were included if they assessed the L-scar technique in breast procedures, including reduction mammaplasty, mastopexy and augmentation, and reported on postoperative complications or patient satisfaction. Searches were performed in PubMed, Embase, Scopus, Web of Science, and Cochrane through December 2024. Pooled prevalences were calculated using a random-effects model, and heterogeneity was assessed via the I^2^ statistic. Risk of bias was evaluated using ROBINS-I.

**Results:**

Twelve studies comprising 3,130 patients were included. The pooled incidence of complications was low across most outcomes: breast asymmetry (1.95%), dog-ear deformity (5%), scar hypertrophy (1.7%), wound dehiscence (1.8%), seroma (0.6%), hematoma (1.2%), infection (0.6%), fat necrosis (5.8%), partial nipple areolar complex (NAC) necrosis (0.4%), and no reported cases of complete NAC necrosis. Sensory complications included nipple sensation loss (2.2%) and reduction (20%). Patient satisfaction was high, with a pooled rate of 92%. All estimates include 95% confidence intervals. Heterogeneity was moderate to high for some outcomes, but sensitivity analyses confirmed the robustness of most estimates.

**Conclusions:**

The L-scar technique demonstrates a favorable safety profile, minimal visible scarring, and excellent satisfaction rates. It appears to be a safe and effective option within the spectrum of contemporary breast reshaping techniques. Further prospective studies are warranted to validate long-term outcomes.

**Level of Evidence III:**

This journal requires that authors assign a level of evidence to each article. For a full description of these Evidence-Based Medicine ratings, please refer to the Table of Contents or the online Instructions to Authors www.springer.com/00266.

**Supplementary Information:**

The online version contains supplementary material available at 10.1007/s00266-026-05869-1.

## Introduction

Breast reshaping surgeries are among the most frequently performed aesthetic surgical procedures worldwide, reflecting the increasing global demand for surgical approaches that combine functional preservation with high patient satisfaction [1, 2]. These procedures present several challenges, including the need to maintain the vascular supply and sensitivity of the nipple areolar complex (NAC), preserve breastfeeding capacity, and achieve aesthetically pleasing, long-lasting results [1]. Although short scar methods have been developed to address some of these concerns, they are often considered suitable only for small to medium volume breast reductions [3].

Multiple incision patterns have been described in breast surgery, including vertical, periareolar, inverted T, and L-shaped designs. Among them, the inverted T technique remains the most widely adopted due to its predictability and consistent outcomes [4]. However, the horizontal component of the scar, particularly its medial segment along the inframammary fold, is often visible and a frequent source of patient dissatisfaction [4]. The L scar technique, described since the 20th century, involves resecting base tissue with superior transposition of the NAC, aiming to reduce scar burden while allowing adequate glandular reshaping and pedicle selection to preserve vascularity and function [5]. By removing tissue primarily from the middle and lower portions of the breast while preserving the lateral neurovascular pedicle, sensitivity may be maintained when appropriate pedicle selection and careful neurovascular handling are employed [6, 7]. Although this classical description forms the historical basis of the technique, subsequent publications have introduced variations in pedicle selection and glandular handling while maintaining the same external L-shaped incision pattern. It is a versatile technique that can be used for correction of breast hypertrophy, ptosis, and asymmetries [8]. Despite benefits, it has not achieved widespread adoption, mainly due to the complexity of preoperative planning and limited reproducibility [4]. However, recent adaptations of the L scar technique have renewed interest in its potential benefits, and studies involving this approach have reported promising, though sometimes controversial, outcomes [4].

This systematic review and meta-analysis aim to synthesize current evidence on the efficacy and safety of the L scar incision technique in breast surgeries, including reduction mammaplasty, mastopexy, and augmentation procedures.

## Methods

This study was conducted and reported in accordance with the Cochrane Collaboration Handbook for Systematic Reviews of Interventions and the Preferred Reporting Items for Systematic Reviews and Meta-Analysis (PRISMA) guidelines [9, 10]. The protocol for this review was prospectively registered with the International Prospective Register of Systematic Reviews (PROSPERO) under registration number CRD420251047389.

### Eligibility Criteria

Studies meeting the following criteria were included: (1) involved patients undergoing breast reshaping procedures, including reduction mammaplasty, mastopexy, or augmentation; (2) evaluated a breast procedure in which the external incision pattern was described by the authors as an L-scar, short lateral scar, or L-shaped incision, irrespective of the internal pedicle (e.g., superior, superomedial, multiplane) or glandular resection pattern; (3) reported any outcomes relevant to this analysis; (4) were designed as randomized controlled trials (RCTs) or observational cohorts; (5) were published in English. Exclusion criteria included: (1) case reports, review articles, editorials, or letters; (2) studies published only as abstracts or conference proceedings; (3) studies that did not evaluate the L-scar technique specifically; (4) studies that failed to report relevant clinical or aesthetic outcomes.

### Data Sources and Search Strategy

PubMed, Embase, Web of Science, Scopus, and Cochrane databases were systematically searched, with the most recent update in December 2024. The complete search strategy was as follows: ("Breast surgery" OR Mammaplasty OR "Mammoplasties" OR "Mammoplasty" OR "Mastopexy" OR “Mastopexies” OR "Breast reconstruction" OR “Breast reconstructions”) AND ("Short scar" OR "L-shaped" OR "L-scar" OR "L-technique" OR "L short scar" OR "lateral scar"). A manual search was conducted for additional eligible publications through the reference lists of all included studies and relevant systematic reviews. Records were managed using the Rayyan® platform (https://www.rayyan.ai/). [11] Two reviewers (P.M. and M.G.) independently and in duplicate screened titles, abstracts, and full texts to determine eligibility. Any conflicts were resolved through discussion and consensus.

### Data Extraction

Data extraction was independently conducted by two authors (L.F. and M.M.), who gathered key information from each study, including publication year, study design, sample size, patient age, and relevant outcomes. For studies with missing summary statistics, we used imputation methods in accordance with Cochrane guidelines and standardized any non-uniform units of measurement. Results were organized and visually displayed using spreadsheets to support systematic synthesis and comparison across studies. [10]

### Outcomes

The outcomes of interest in this review included both clinical complications and patient-reported measures related to the use of the L-scar technique in mammaplasty. Specifically, the following outcomes were analyzed: partial and complete necrosis of the NAC, scar hypertrophy, wound dehiscence, seroma formation, breast asymmetry, presence of dog-ears, hematoma, fat necrosis, infection, nipple sensation loss, nipple sensation reduction, and overall patient satisfaction.

Partial NAC necrosis was defined as localized tissue ischemia resulting in partial loss of viability of the areolar complex, while complete NAC necrosis referred to full-thickness tissue death requiring further intervention [12]. Scar hypertrophy refers to the formation of excessive scar tissue beyond the expected healing process [13]. Wound dehiscence was characterized by the reopening of the surgical incision, and seroma formation referred to the accumulation of fluid at the surgical site [14, 15]. Breast asymmetry refers to any noticeable difference in volume, contour, or position between the two breasts after surgery [16]. Dog-ears were defined as lateral protrusions or puckering at the ends of the incision [17]. Hematoma was defined as postoperative blood collection under the skin [18]. Fat necrosis involves the formation of firm nodules due to localized adipose tissue death [19]. Infection was identified based on clinical or microbiological evidence of inflammation at the surgical site [20]. Nipple sensation loss refers to complete absence of sensory perception in the NAC, while reduction indicates a partial decrease in sensitivity [21]. Patient satisfaction was evaluated as a subjective measure of the patient’s perception of aesthetic and functional outcomes, as reported in the original studies [22]. Measurement methods varied considerably and included telephone-administered questionnaires, study-specific satisfaction scales (e.g., categorical A–C scales), and informal, non-validated surveys conducted during follow-up visits. Anonymity of responses was not consistently described.

### Quality Assessment

The methodological quality and risk of bias of included non-randomized studies were evaluated using the ROBINS-I tool [23]. Two reviewers (L.C. and P.M.) performed the assessments independently, with discrepancies resolved through discussion. Studies were classified as having low, moderate, or high risk of bias based on overall domain evaluations.

### Statistical Analysis

This was a single-arm meta-analysis estimating the pooled prevalence of each outcome or complication in patients undergoing L-scar breast surgery. A single-proportion analysis with 95% confidence intervals (CIs) was conducted under a random-effects model.

Pooled estimates were calculated using the inverse variance method, complemented by both DerSimonian and Laird and restricted maximum likelihood (REML) estimators, depending on model fit and outcome characteristics [24]. Heterogeneity was assessed using the I^2^ statistic, with thresholds of 0–40% considered low, 40–60% moderate, and above 60% high [25]. Visual tools, including forest plots and Bajaut plots, were used to explore between-study variability. To further address substantial heterogeneity, sensitivity analyses were performed by systematically excluding one study at a time and recalculating the pooled results. All statistical analyses were conducted using R software (version 4.4.2; R Foundation for Statistical Computing, Vienna, Austria), employing the Meta and Metafor packages. [26]

## Results

**Fig. 1:**
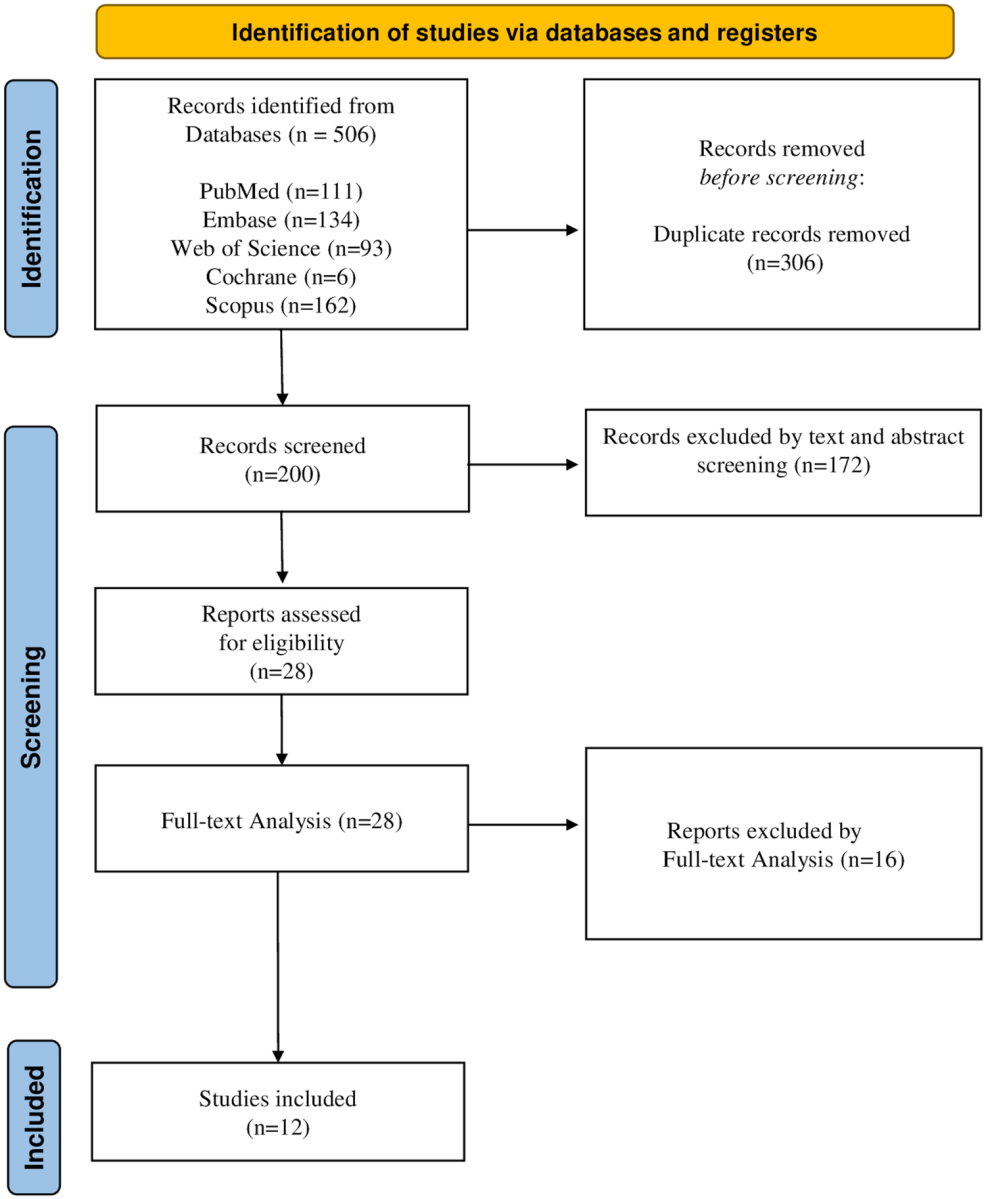
PRISMA flow diagram of study screening and selection process.

Our search identified 506 potentially relevant records from five databases. After removing 306 duplicates, 200 studies were screened by title and abstract. Of these, 165 were excluded for being irrelevant to the topic or lacking outcome data. Twenty-eight full-text articles were assessed for eligibility, and 16 were excluded due to insufficient reporting of outcomes or lack of evaluation of the L-scar technique, resulting in 12 studies included in the final analysis (Fig. 1) [1, 3–6, 27–33]. Patients’ number per study ranged from 45 to 1,217, with a total pooled sample of 3,130 individuals who underwent mastopexy, reduction mammaplasty, or augmentation procedures using the L-scar approach. As summarized in Table 1, most studies were retrospective in design and conducted primarily in Brazil, the United States, and Germany. Follow-up durations ranged from 5 to 141 months, with patient ages between 16 and 73 years. Surgical strategies and reporting of implant type and resected tissue volume varied across studies, reflecting methodological heterogeneity in operative techniques and outcome documentation.

**Table 1:** Baseline characteristics of included studies

Author, Year Study Period	Patients (N)	Study Design	Country	Surgery Type, Patients (N)	Age, years (Mean ± SD) [Median IQR] or {Range}	Follow-up, months (Mean ± SD) [Median IQR] or {Range}	Implant Type	Resected Tissue, grams (Mean ± SD) [Median IQR] or {Range}
Akyurek, 2015 (2008-2014)	69	R	USA	MM: 69	38 {16-68}	10 {2-82}	NA	1333.8 {1002-3275}
Bark, 2023 (2016-2021)	632	R	Brazil	AMT: 632	38 {18-71}	12-60	Round, nanotextured implants	117.45 {5-530}
Bark, 2024 (2021-2022)	213	R	Brazil	MT: 213	36.6 {18-62}	6-24	NA	NA
Chiari, 2011 (NA period)	64	P	Brazil	MT and RMM: 64	35.2 {16-59}	12	NA	214.1 ± 172.4 [0 - 1000]
Coelho, 2004 (1996-2003)	500	R	Brazil	MT and RMM: 500	NA	NA	NA	500 [200-1200]
Mcculley, 1999 (1993-1997)	90	R	South Africa	MM: 90	NA	At least 5	NA	686 {210-2500}
Munhoz, 2023 (2017-2021)	45	R	Brazil	AMT: 45	37.7 ± 7.2	38 {6-49}	Smooth Silk Surface Round Implants	175 ^6^
Pallua, 2003 (1999-2001)	45	R	Germany	MM: 45	36 (NA)	13 {6-17}	NA	625 {200-2080}
Pallua, 2020 (2005-2016)	60	R	Germany	RMM: 60	43.82 ± 2.08	12	NA	NA
Pechter, 2008 (2004-2007)	72	R	USA	MT: 14; AMT: 52; Implant Removal/Replacement + MT: 6​	41 {18-66}	NA	NA	NA
Sodré, 2021 (2011-2021)	123	R	Brazil	AMT: 123	35.6 {19-64}	24	Textured, Round, Cohesive Silicone Gel	NA
Wall, 2023 (2005-2015)	1217	R	USA	AMT:1217	36 {14-73}	39 {4-141}	Silicone and Saline	NA

*R* Retrospective, *P* Prospective, *MM* Mammaplasty, *RMM* Reduction mammaplasty, *MT* Mastopexy, *AMT* Augmentation mastopexy, *SD* Standard deviation, *IQR* Interquartile range, *NA* Not available/applicable

### Pooled Analysis of Included Studies

#### Patient Satisfaction

**Fig. 2:**
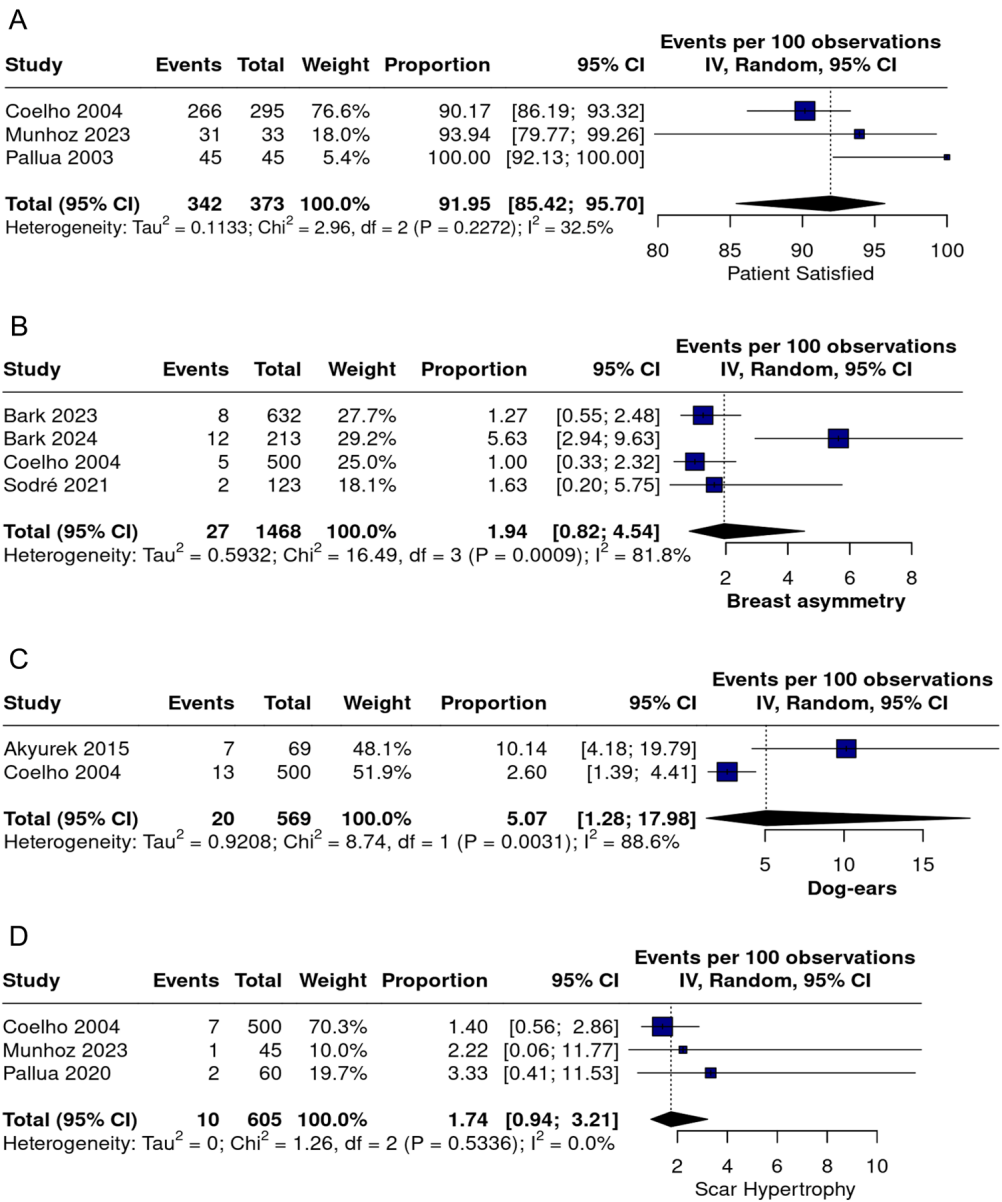
**A**: Patient satisfaction forest plot; **B**: Breast asymmetry forest plot; **C**: Dog-ears forest plot; **D**: Scar hypertrophy forest plot

In a meta-analysis of 3 studies, the proportion of satisfied patients was 92% (95% CI 85.4–95.7; I^2^ = 32.5%, Fig. 2A), indicating high satisfaction with low to moderate heterogeneity.

#### Breast Asymmetry

In a meta-analysis of 4 studies, the pooled incidence of breast asymmetry was 1.95% (95% CI, 0.82–4.54; I^2^ = 81.8%, Fig. 2B), indicating substantial heterogeneity.

#### Dog-ears Deformity

In a meta-analysis of 2 studies, the pooled incidence of dog-ear deformity was 5% (95% CI 1.28–17.98; I^2^ = 88.6%, Fig. 2C), with considerable heterogeneity observed.

#### Scar Hypertrophy

In a meta-analysis of 3 studies, the pooled incidence of scar hypertrophy was 1.7% (95% CI 0.94–3.21; I^2^ = 0%, Fig. 2D), with no heterogeneity detected.

#### Wound Dehiscence

**Fig. 3:**
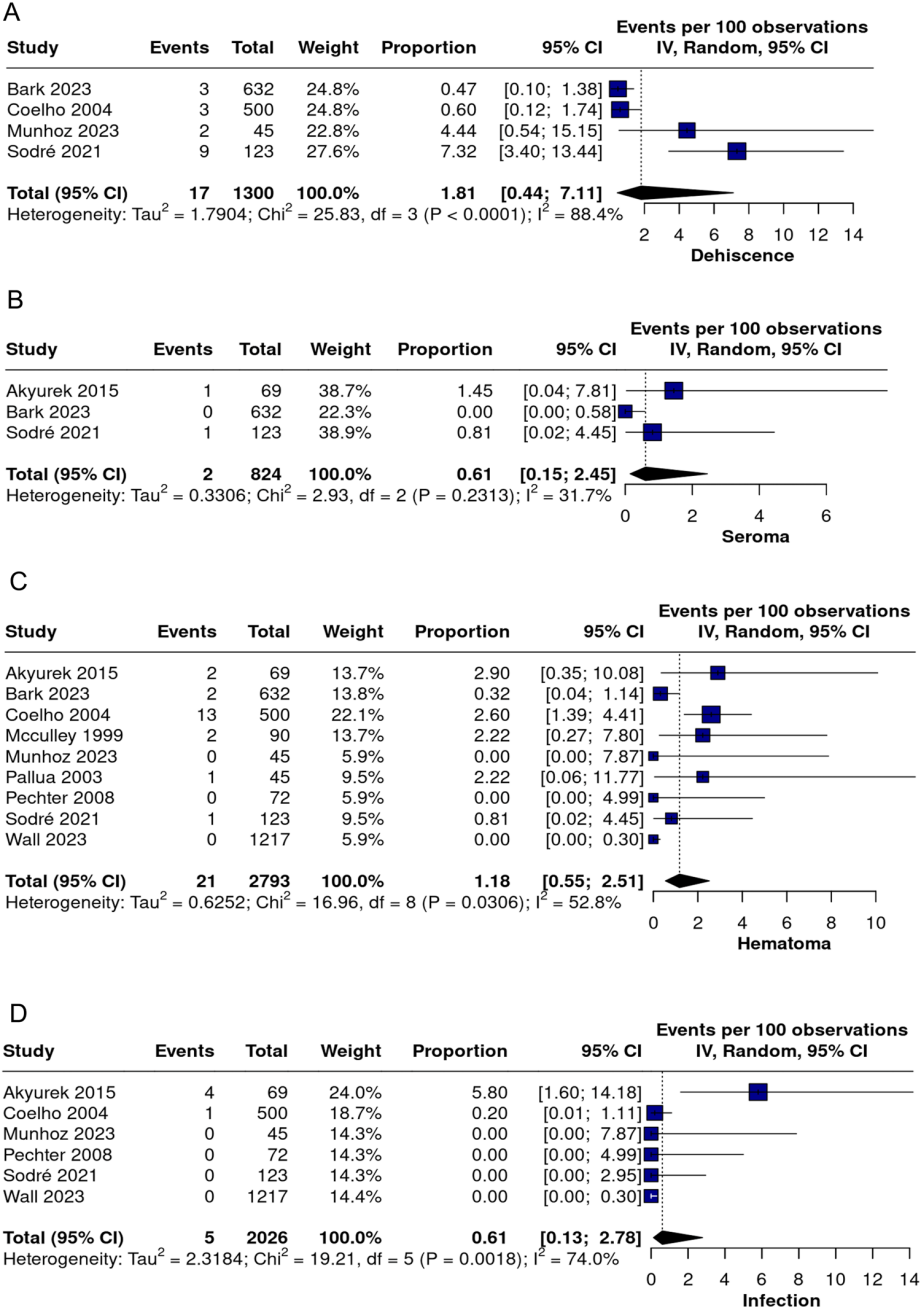
**A**: Wound dehiscence forest plot; **B**: Seroma formation forest plot; **C**: Hematoma forest plot; **D**: Infection forest plot

In a meta-analysis of 4 studies, the pooled incidence of wound dehiscence was 1.8% (95% CI 0.44–7.11; I^2^ = 88.4%, Fig. 3A), with substantial heterogeneity across studies.

#### Seroma Formation

In a meta-analysis of 3 studies, the pooled incidence of seroma formation was 0.6% (95% CI, 0.15–2.45; I^2^ = 31.7%, Fig. 3B), indicating moderate heterogeneity.

#### Hematoma

In a meta-analysis of 9 studies, the pooled incidence of hematoma was 1.2% (95% CI, 0.55–2.51; I^2^ = 52.8%, Fig. 3C), indicating moderate heterogeneity among the studies.

#### Infection

In a meta-analysis of 6 studies, the infection rate was 0.6% (95% CI 0.1–2.8; I^2^ = 74.0%, Fig. 3D), with high heterogeneity. Most studies reported no events.

#### Nipple-Areolar Complex Partial Necrosis

**Fig. 4:**
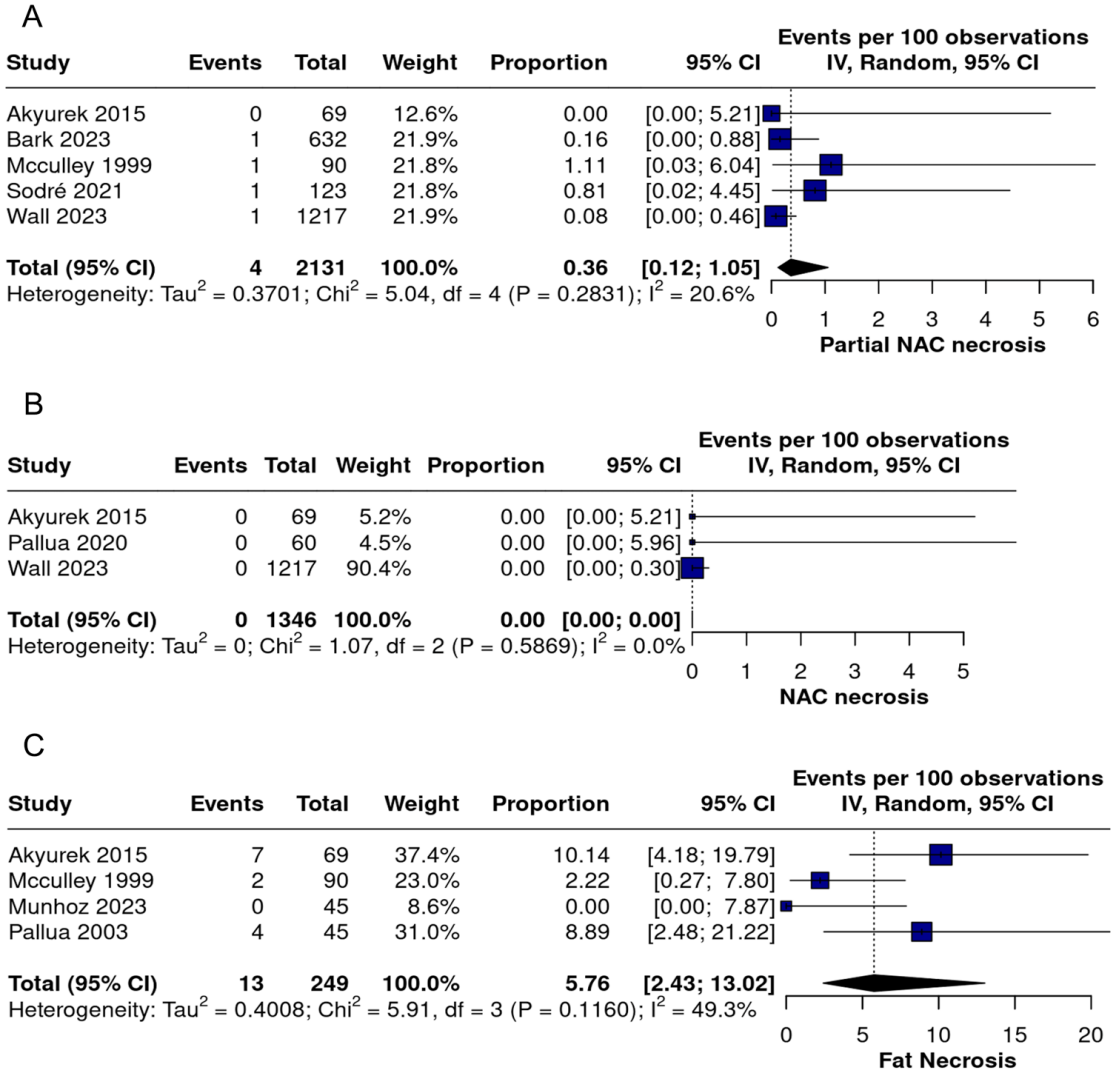
**A**: Nipple-Areolar complex partial necrosis forest plot; **B**: Complete nipple-areolar complex necrosis forest plot; **C**: Fat necrosis forest plot

In a meta-analysis of 5 studies, the pooled rate of partial NAC necrosis was 0.4% (95% CI 0.12–1.05; I^2^ = 20.6%, Fig. 4A), with low heterogeneity.

#### Complete Nipple-Areolar Complex Necrosis

In a meta-analysis of 3 studies, there were no reported cases of complete NAC necrosis. The pooled incidence was 0% (95% CI 0.00–0.00; I^2^ = 0%, Fig. 4B), indicating no heterogeneity.

#### Fat Necrosis

In a meta-analysis of 4 studies, the rate of fat necrosis was 5.8% (95% CI 2.4–13.0; I^2^ = 49.3%, Fig. 4C), with moderate heterogeneity across studies.

#### Nipple Sensation Loss

**Fig. 5:**
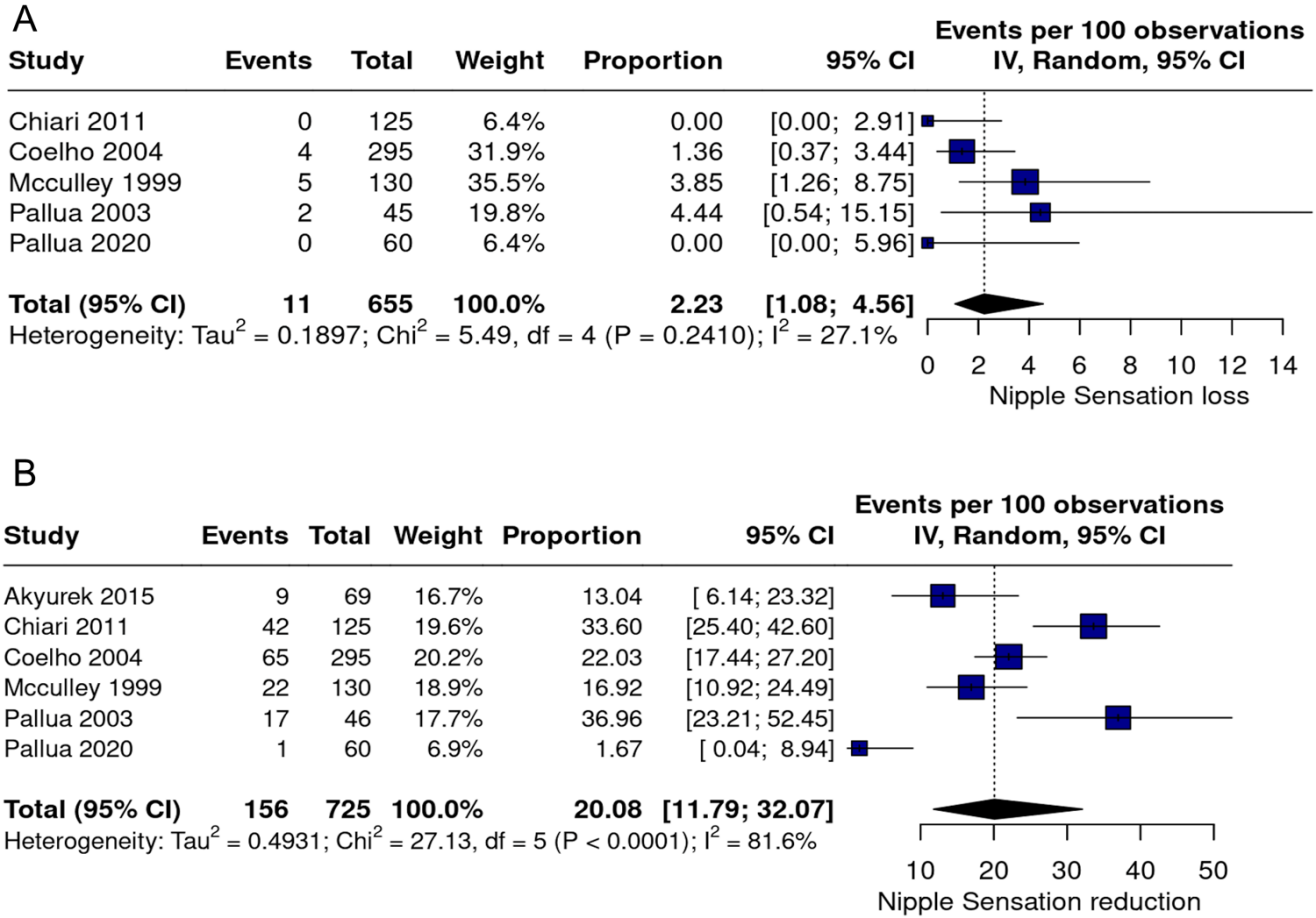
**A**: Nipple sensation loss forest plot; **B**: Nipple sensation reduction forest plot

In a meta-analysis of 5 studies, the rate of complete nipple sensation loss was 2.2% (95% CI 1.1–4.6; I^2^ = 27.1%, Fig. 5A), with low heterogeneity.

#### Nipple Sensation Reduction

**Fig. 6:**
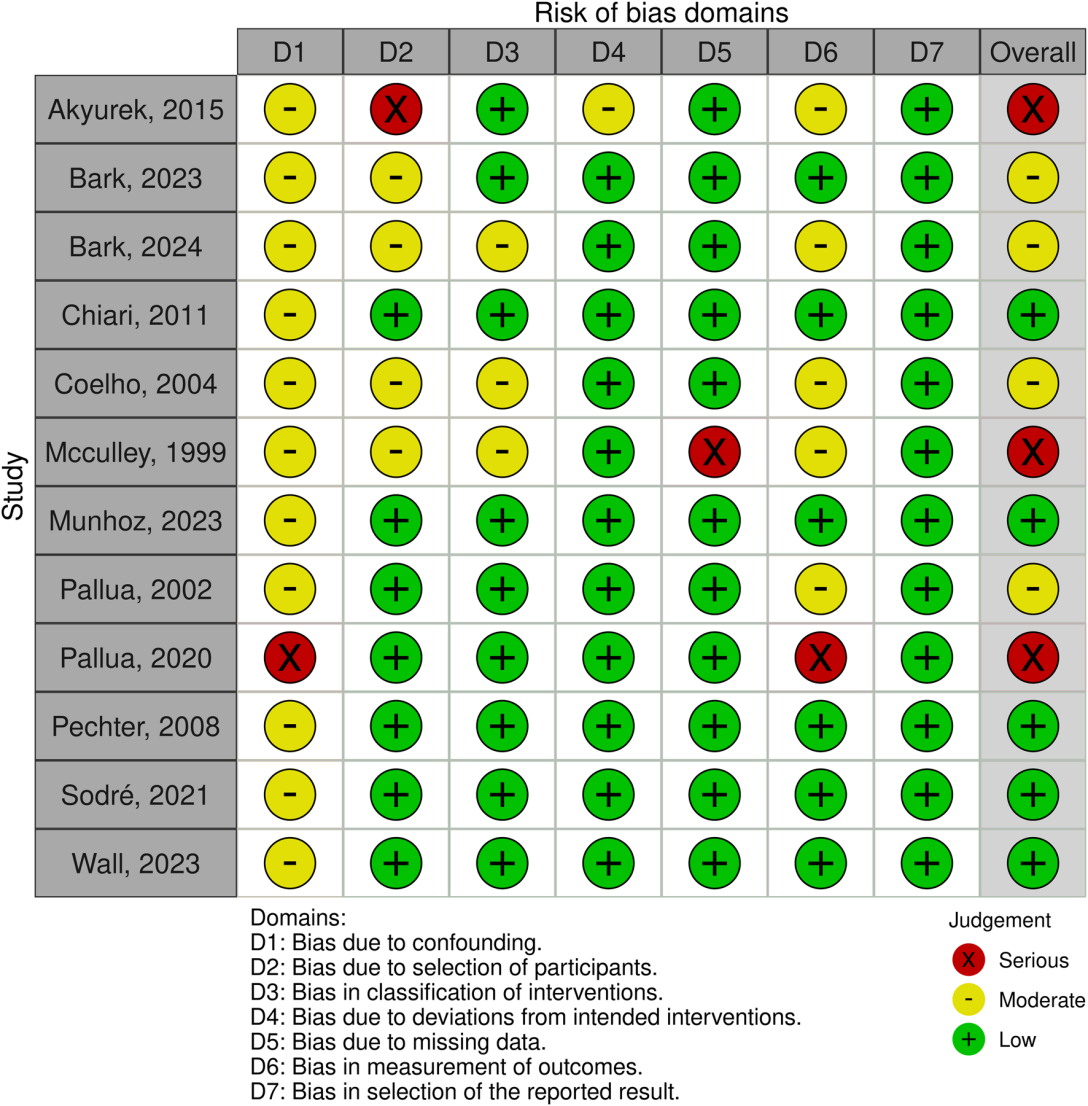
Risk of bias of the included studies

In a meta-analysis of 6 studies, the rate of nipple sensation reduction was 20% (95% CI 11.8–32.1; I^2^ = 81.6%, Fig. 5B), with substantial heterogeneity between studies (Fig. 6).

### Sensitivity Analysis

To address heterogeneity, sensitivity analyses were conducted using the leave-one-out method. For fat necrosis, omitting Mcculley 1999 resulted in the lowest heterogeneity (I^2^ = 20%, 9% [95% CI: 5–15%]) (Supplementary Figure 1). For infection, the lowest heterogeneity was observed when omitting Akyurek 2015 (I^2^ = 0%, 0.3% [95% CI: 0.09–0.87]) (Supplementary Figure 2). For nipple sensation reduction, the lowest heterogeneity was found when omitting Coelho 2004 (I^2^ = 78%, 18% [95% CI: 8–36%]) (Supplementary Figure 3). For hematoma, omitting Wall 2023 resulted in the lowest heterogeneity (I^2^ = 28%, 2% [95% CI: 1–3%]) (Supplementary Figure 4). For breast asymmetry, the lowest heterogeneity occurred when omitting Bark 2024 (I^2^ not reported, 1.2% [95% CI: 0.73–2.00]) (Supplementary Figure 5).

### Risk of Bias Assessment

The risk of bias of the included non-randomized studies was assessed using the ROBINS-I tool. Three studies [1, 3, 28] were rated as having a serious overall risk of bias, while four studies [4, 5, 27, 30] were judged to have a moderate overall risk. The remaining five studies [6, 29, 31–33] were considered to have low overall risk.

In summary, the main sources of bias across the included studies were related to confounding, selection of participants, and measurement of outcomes, while all studies demonstrated low risk of reporting bias.

## Discussion

Our meta-analysis consolidates the growing evidence supporting the L-scar approach as a versatile and effective technique for both reduction mammaplasty and mastopexy. Synthesizing outcomes from more than 3,100 patients, our findings reaffirm the clinical viability of this method, particularly when evaluating its complication profile and patient satisfaction outcomes.

Patient satisfaction was remarkably high at 92%, emerging as one of the most compelling outcomes of this meta-analysis. This finding underscores the technique’s ability to meet patients' aesthetic and psychosocial expectations, a critical benchmark in elective breast surgery [34, 35]. Notably, Saariniemi et al., in a RCT, observed that even with objectively similar complication rates, patient satisfaction is often more tightly linked to aesthetics and scarring than to minor clinical events. [36]

The pooled incidence of breast asymmetry was low (1.9%), suggesting a high degree of surgical precision and effective tissue handling in L-scar procedures. These results align with the retrospective cohort by Morrison et al., which highlighted symmetry preservation as a central strength of short-scar techniques in adolescent populations undergoing breast reduction [37]. Additionally, the long-term follow-up study by Hilewitz et al. confirmed the maintenance of breast shape and volume, reinforcing the L-scar’s aesthetic sustainability. [38]

A particularly relevant concern in aesthetic breast surgery is dog-ear deformity, which we found to occur in 5% of cases. Although higher than other complications, this rate remains within acceptable surgical ranges. Grassetti et al. proposed a 90° incision technique as a refinement for dog-ear correction without lengthening the scar, offering a potential adjunct to L-scar procedures where such deformities persist [39]. Similarly, El Hajj et al. demonstrated the effectiveness of an L-shaped incision combined with lipoaspiration in avoiding lateral dog ears after mastectomy, providing surgical rationale for the geometric advantages of this pattern. [40]

The incidence of scar hypertrophy was low (1.7%). This may reflect both careful patient selection and intrinsic advantages of the L-scar’s tension distribution. A RCT by Thoma et al. comparing vertical and inverted-T reductions suggested that reduced scar burden can lead to improved patient-reported outcomes in quality-of-life metrics [41]. Our findings indirectly support this, as hypertrophic scarring remains a key determinant of long-term satisfaction.

Wound dehiscence, with a rate of 1.8%, remains a complication to monitor, particularly in high-risk patients. Mu et al. described a double-ring breast reduction design that aims to minimize wound tension across junction points, which may offer insight into further refinements of L-scar closures. [42]

Seroma formation (0.6%) and hematoma (1.2%) presented low-to-moderate pooled incidences, consistent with what is typically expected for mastopexies and reductions. Despite minor inter-study heterogeneity, these results confirm that the L-scar approach does not confer an elevated risk for fluid collections or bleeding. Morrison et al.’s complication audit in adolescent reductions supports these findings, reporting comparable or higher rates in traditional techniques. [37]

Infection was rare (0.6%) across the pooled studies, despite moderate heterogeneity (I^2^ = 74%). This low incidence supports the safety of the technique, though patient comorbidities and perioperative protocols may also contribute to outcome variability.

Regarding the NAC, partial necrosis was extremely uncommon (0.4%) and complete necrosis was not reported in any included study (0%). These findings suggest that the pedicle designs employed in these procedures were associated with preserved NAC viability. Wall et al. reported similar rates in their 10-year review of short-scar mastopexies, reinforcing the reliability of superiorly based pedicles in maintaining NAC viability. [33]

Fat necrosis occurred in 5.8% of cases. While relatively higher, this figure is compatible with expectations in glandular reductions. Munhoz et al. reported that combining fat grafting with L-scar augmentation strategies can both improve contour and potentially reduce necrosis by optimizing vascular distribution. [29]

Finally, the rates of nipple sensation loss (2.2%) and reduction (20%) are essential for evaluating patient-centered outcomes. Although reduction in nipple sensation was not uncommon, most patients experience partial changes rather than complete loss. A meta-analysis by Li et al. demonstrated comparable results in vertical versus inverted-T techniques, with the L-scar falling within an acceptable complication spectrum. [43]

The L-scar technique, by reducing scar length without compromising safety, may help optimize this balance. This aligns with findings from Iwuagwu et al., who, RCT, demonstrated that bilateral reduction mammaplasty significantly improves patients’ quality of life and emotional well-being, reinforcing that aesthetic and psychosocial outcomes are major drivers of postoperative satisfaction in elective breast surgery. [44]

## Limitations

This meta-analysis has limitations. First, although over 3,000 patients were included, only twelve studies met the strict eligibility criteria. This relatively limited number reflects both the specificity of the L-scar incision pattern as an isolated surgical variable and the absence of standardized outcome reporting in many breast surgery publications, which precluded their inclusion. Most available studies were observational in design, and only a few provided long-term follow-up, limiting the ability to assess sustained satisfaction and late complications. The predominance of retrospective designs further limits causal inference regarding the independent role of incision pattern on outcomes.

Second, heterogeneity was considerable in some outcomes (for instance, nipple sensation reduction: I^2^ = 82%), suggesting variability in definitions, surgical techniques, and reporting standards. Many studies did not clearly define the outcomes assessed, which may have introduced additional inconsistency. Additionally, revision or secondary correction rates were not consistently reported across the included studies, precluding pooled analysis of this clinically relevant endpoint. Given that short-scar techniques may occasionally require secondary refinements, underreporting of revision procedures may represent an additional source of bias and may influence interpretation of satisfaction outcomes. Furthermore, patient satisfaction was assessed using heterogeneous and predominantly non-validated instruments, often without standardized reporting of response rates or anonymity, which may have introduced response and reporting bias.

Third, due to reporting gaps and heterogeneity in surgical technique descriptions, subgroup analyses could not be performed according to surgical indication (reduction, mastopexy, augmentation) or pedicle type (e.g., superior, superomedial, inferior). Variability in pedicle selection and glandular remodeling techniques across studies may have influenced complication and satisfaction outcomes. In addition, in reduction cases, surgeons may have selectively avoided using short-scar patterns in patients with very large breast volumes or severe ptosis, where longer incision patterns are often preferred. This potential selection bias may have influenced the observed complication rates and satisfaction outcomes.

To mitigate these limitations, we applied random-effects models, conducted sensitivity analyses, and systematically evaluated the risk of bias across all included studies.

## Conclusion

The L-scar technique demonstrates a favorable safety profile, low complication rates, and high patient satisfaction. Although direct comparative analyses with other incision patterns were not performed, the pooled outcomes observed fall within a favorable range when contextualized within the existing literature. These findings suggest that the L-scar represents a safe and reliable option in appropriately selected patients undergoing breast reshaping procedures. Future large prospective studies and RCTs are warranted to further validate long-term outcomes and comparative effectiveness.

## Supplementary Information

Below is the link to the electronic supplementary material.

Supplementary file1 (TIFF 21447 KB)

Supplementary file2 (TIFF 24379 KB)

Supplementary file3 (TIFF 26087 KB)

Supplementary file4 (TIFF 28905 KB)

Supplementary file5 (TIFF 28450 KB)

## Data Availability

All authors take responsibility for all aspects of the reliability and freedom from bias of the data presented and their discussed interpretation.

## References

[1] Pallua N, Kim B-S, O’Dey DM. The short scar three-block L-wing technique. J Plast Reconstr Aesthet Surg. 2020;73(6):1075–80. 10.1016/j.bjps.2020.01.027. 32317232 10.1016/j.bjps.2020.01.027

[2] Triana L, Palacios Huatuco RM, Campilgio G, Liscano E. Correction: trends in surgical and nonsurgical aesthetic procedures: a 14-year analysis of the International Society of Aesthetic Plastic Surgery-ISAPS. Aesthetic Plast Surg. 2024;48(21):4601. 10.1007/s00266-024-04355-w. 39225818 10.1007/s00266-024-04355-w

[3] Akyurek M, Chappell AG. Short-scar mammaplasty in severe macromastia. Ann Plast Surg. 2016;77(6):609–14. 10.1097/SAP.0000000000000678. 26678101 10.1097/SAP.0000000000000678

[4] Bark AA Jr., Minikowski GC, Mujahed IBU. Multiplane L-scar augmentation mastopexy: an individualized approach to muscle, glandular tissue, and skin. Plast Reconstr Surg. 2024;153(4):801–9. 10.1097/PRS.0000000000010850. 37335763 10.1097/PRS.0000000000010850

[5] Coelho de Almeida CI. Mammaplasty with L-incision. Aesthet Surg J. 2004;24(2):102–11. 10.1016/j.asj.2003.12.006. 19336144 10.1016/j.asj.2003.12.006

[6] Chiari A Jr., Nunes TA, Grotting JC, Cotta FB, Gomes RCB. Breast sensitivity before and after the L short-scar mammaplasty. Aesthetic Plast Surg. 2012;36(1):105–14. 10.1007/s00266-011-9756-2. 21638163 10.1007/s00266-011-9756-2

[7] Richard L, Delay E, Payement G, Cresseaux P, Cantaloube D. Mammaplasty with an L-shaped scar and a pre-established design. Apropos of 80 cases. Ann Chir Plast Esthet. 1998;43(1):69–77. 9768095

[8] Hall-Findlay EJ. A simplified vertical reduction mammaplasty: shortening the learning curve. Plast Reconstr Surg. 1999;104(3):748–59. 10.1097/00006534-199909010-00020. (**discussion 760-3**). 10456528

[9] Cumpston M, Li T, Page MJ, et al. Updated guidance for trusted systematic reviews: a new edition of the Cochrane Handbook for Systematic Reviews of Interventions. Cochrane Database Syst Rev. 2019;10:ED000142. 10.1002/14651858.ED000142. 31643080 10.1002/14651858.ED000142 PMC10284251

[10] Page MJ, McKenzie JE, Bossuyt PM, et al. The PRISMA 2020 statement: an updated guideline for reporting systematic reviews. Rev Esp Cardiol (Engl Ed). 2021;74(9):790–9. 10.1016/j.rec.2021.07.010. 34446261 10.1016/j.rec.2021.07.010

[11] Ouzzani M, Hammady H, Fedorowicz Z, Elmagarmid A. Rayyan—a web and mobile app for systematic reviews. Syst Rev. 2016. 10.1186/s13643-016-0384-4. 27919275 10.1186/s13643-016-0384-4 PMC5139140

[12] Perez-Otero S, Hemal K, Boyd CJ, et al. Minimizing nipple-areolar complex complications in prepectoral breast reconstruction after nipple-sparing mastectomy. Ann Plast Surg. 2024;92(4S Suppl 2):S179–84. 10.1097/SAP.0000000000003906. 38556670 10.1097/SAP.0000000000003906

[13] Brissett AE, Sherris DA. Scar contractures, hypertrophic scars, and keloids. Facial Plast Surg. 2001;17(4):263–72. 10.1055/s-2001-18827. 11735059 10.1055/s-2001-18827

[14] Muller-Sloof E, de Laat E, Zwanenburg P, et al. Exploring the definition of surgical wound dehiscence in literature: a scoping review. J Tissue Viabil. 2024;33(4):923–9. 10.1016/j.jtv.2024.09.006. 10.1016/j.jtv.2024.09.006 39341772

[15] Srivastava V, Basu S, Shukla VK. Seroma formation after breast cancer surgery: what we have learned in the last two decades. J Breast Cancer. 2012;15(4):373–80. 10.4048/jbc.2012.15.4.373. 23346164 10.4048/jbc.2012.15.4.373 PMC3542843

[16] Onesti MG, Mezzana P, Martano A, Scuderi N. Breast asymmetry: a new vision of this malformation. Acta Chir Plast. 2004;46(1):8-11. 15274471

[17] Kauhanen S, Höckerstedt A. Full breast reconstruction with fat and how to recycle the “dog-ear.” Gland Surg. 2019;8(Suppl 4):S297–300. 10.21037/gs.2019.05.07. 31709171 10.21037/gs.2019.05.07 PMC6819887

[18] Hashmi DL, Ong AW, Muller A, Itzoe M, Martin A, Foster SM. Breast hematoma: an under-recognized and under-reported female-specific traumatic injury and its clinical significance. Am Surg. 2021;87(1):156–8. 10.1177/0003134820943568. 32902302 10.1177/0003134820943568

[19] Chang CS, Lanni MA, Mirzabeigi MN, Bucky LP. Large-volume fat grafting: identifying risk factors for fat necrosis. Plast Reconstr Surg. 2022;150(5):941e–9e. 10.1097/PRS.0000000000009655. 35993869 10.1097/PRS.0000000000009655

[20] Costanzo D, Romeo A. Surgical site infections in breast surgery - A primer for plastic surgeons. Eplasty. 2023;23:e18. PMC10176482 37187867

[21] Nahabedian MY, Mofid MM. Viability and sensation of the nipple-areolar complex after reduction mammaplasty. Ann Plast Surg. 2002;49(1):24–31. 10.1097/00000637-200207000-00004. (**discussion 31-2**). 12142591 10.1097/00000637-200207000-00004

[22] Batenburg MCT, Gregorowitsch ML, Maarse W, et al. Patient-reported cosmetic satisfaction and the long-term association with quality of life in irradiated breast cancer patients. Breast Cancer Res Treat. 2020;179(2):479-489. 10.1007/s10549-019-05470-y 10.1007/s10549-019-05470-y PMC6987082 31650347

[23] Sterne JA, Hernán MA, Reeves BC, et al. ROBINS-I: a tool for assessing risk of bias in non-randomised studies of interventions. BMJ. 2016;355:i4919. 10.1136/bmj.i4919 10.1136/bmj.i4919 PMC5062054 27733354

[24] DerSimonian R, Laird N. Meta-analysis in clinical trials. Control Clin Trials. 1986;7(3):177-188. 10.1016/0197-2456(86)90046-2 10.1016/0197-2456(86)90046-2 3802833

[25] Higgins JPT, Thompson SG, Deeks JJ, Altman DG. Measuring inconsistency in meta-analyses. BMJ. 2003;327(7414):557-560. 10.1136/bmj.327.7414.557 10.1136/bmj.327.7414.557 PMC192859 12958120

[26] Balduzzi S, Rücker G, Schwarzer G. How to perform a meta-analysis with R: a practical tutorial. Evid Based Ment Health. 2019;22(4):153–60. 10.1136/ebmental-2019-300117. 31563865 10.1136/ebmental-2019-300117 PMC10231495

[27] Bark AA, Jr., Minikowski GC, Moreto L, Mujahed IBU. Creating a new inframammary fold, raising the breast footprint, and elongating the torso with a multiplane concept. Plast Reconstr Surg. 2024;154(6):1084e-1090e. 10.1097/PRS.0000000000011265 10.1097/PRS.0000000000011265 38276955

[28] McCulley SJ, Rousseau TE. A modified Chiari L short-scar mammoplasty--the technique and results. Br J Plast Surg. 1999;52(2):112-117. 10.1054/bjps.1998.3051 10.1054/bjps.1998.3051 10434889

[29] Munhoz AM, Marques Neto AdA, Maximiliano J. Hybrid augmentation mastopexy with new generation of smooth surface implants: Combining the benefits of fat grafting, inferior muscle support, and an L-shaped scar. Plast Reconstr Surg. 2023;152(1):29e-41e. 10.1097/PRS.0000000000010196 10.1097/PRS.0000000000010196 36728268

[30] Pallua N, Ermisch C. "I" becomes "L": modification of vertical mammaplasty. Plast Reconstr Surg. 2003;111(6):1860-1870. 10.1097/01.PRS.0000056870.72470.A7 10.1097/01.PRS.0000056870.72470.A7 12711945

[31] Pechter EA, Roberts S. The versatile helium balloon mastopexy. Aesthet Surg J. 2008;28(3):272-278. 10.1016/j.asj.2008.03.002 10.1016/j.asj.2008.03.002 19083537

[32] Sodré RL, Calil JA, Ferreira RdA, et al. The L-shaped mastopexy. Rev Bras Cir Plást. 2023;38(2)10.5935/2177-1235.2023rbcp0684-en

[33] Wall SH, Claiborne JR, Wall HC. Superiorly based short-scar mastopexy augmentation: A 10-year review of 1217 consecutive cases. Plast Reconstr Surg. 2023;151(6):918e-930e. 10.1097/PRS.0000000000010129 10.1097/PRS.0000000000010129 36728622

[34] Graf R, Biggs TM, Steely RL. Breast shape: a technique for better upper pole fullness. Aesthetic Plast Surg. 2000;24(5):348–52. 10.1007/s002660010057. 11084696 10.1007/s002660010057

[35] Graf R, Biggs TM. In search of better shape in mastopexy and reduction mammoplasty. Plast Reconstr Surg. 2002;110(1):309–17. 10.1097/00006534-200207000-00053. 12087273 10.1097/00006534-200207000-00053

[36] Saariniemi KMM, Keranen UH, Salminen-Peltola PK, Kuokkanen HOM. Reduction mammaplasty is effective treatment according to two quality of life instruments. a prospective randomised clinical trial. J Plast Reconstr Aesthet Surg. 2008;61(12):1472–8. 10.1016/j.bjps.2007.09.024. 17983882 10.1016/j.bjps.2007.09.024

[37] Morrison KA, Vernon R, Choi M, Karp NS. Quantifying surgical complications for reduction mammaplasty in adolescents. Plast Reconstr Surg. 2023;151(3):376e–83e. 10.1097/PRS.0000000000009905. 36730536 10.1097/PRS.0000000000009905

[38] Hilewitz D, Ganor O, Adler N, et al. Long-term breast shape analysis after short-scar reduction mammaplasty: a critical view. Plast Reconstr Surg Glob Open. 2025;13(1):e6428. 10.1097/GOX.0000000000006428. 39810903 10.1097/GOX.0000000000006428 PMC11730089

[39] Grassetti L, Lazzeri D, Torresetti M, Bottoni M, Scalise A, Di Benedetto G. Aesthetic refinement of the dog ear correction: the 90° incision technique and review of the literature. Arch Plast Surg. 2013;40(3):268–9. 10.5999/aps.2013.40.3.268. 23730608 10.5999/aps.2013.40.3.268 PMC3665876

[40] El Hajj H, Millochau J, Mpungu LF, et al. Avoiding post mastectomy lateral “dog ears” by an L incision and lipoaspiration: the Paris Breast Center technique. Eur J Surg Oncol. 2022;48(9):1925–8. 10.1016/j.ejso.2022.05.017. 35688712 10.1016/j.ejso.2022.05.017

[41] Thoma A, Ignacy TA, Duku EK, et al. Randomized controlled trial comparing health-related quality of life in patients undergoing vertical scar versus inverted T-shaped reduction mammaplasty. Plast Reconstr Surg. 2013;132(1):48e–60e. 10.1097/PRS.0b013e3182910cb0. 23806954 10.1097/PRS.0b013e3182910cb0

[42] Mu D, Lin Y, Zhang X, Li Z. Principle and logic of vertical reduction repair double-ring breast reduction. Aesthetic Plast Surg. 2025;49(3):727–32. 10.1007/s00266-024-04346-x. 39227472 10.1007/s00266-024-04346-x

[43] Li Z, Qian B, Wang Z, et al. Vertical scar versus Inverted-T scar reduction mammaplasty: a meta-analysis and systematic review. Aesthetic Plast Surg. 2021;45(4):1385–96. 10.1007/s00266-021-02167-w. 33649925 10.1007/s00266-021-02167-w

[44] Iwuagwu OC, Walker LG, Stanley PW, Hart NB, Platt AJ, Drew PJ. Randomized clinical trial examining psychosocial and quality of life benefits of bilateral breast reduction surgery. Br J Surg. 2006;93(3):291–4. 10.1002/bjs.5234. 16363021 10.1002/bjs.5234

